# Economic Costs of Measles Outbreak in the Netherlands, 2013–2014

**DOI:** 10.3201/eid2111.150410

**Published:** 2015-11

**Authors:** Anita W.M. Suijkerbuijk, Tom Woudenberg, Susan J.M. Hahné, Laura Nic Lochlainn, Hester E. de Melker, Wilhelmina L.M. Ruijs, Anna K. Lugnér

**Affiliations:** National Institute for Public Health and the Environment (RIVM), Bilthoven, the Netherlands (A.W.M. Suijkerbuijk, T. Woudenberg, S.J.M. Hahné, L. Nic Lochlainn, H.E. de Melker, W.L.M. Ruijs, A.K. Lugnér);; European Centre for Disease Prevention and Control, Stockholm, Sweden (L. Nic Lochlainn)

**Keywords:** health care economics, costs, cost analysis, measles, measles-mumps-rubella vaccine, vaccination, primary prevention, disease outbreak, viruses, encephalitis, religion and medicine, the Netherlands

## Abstract

In 2013 and 2014, the Netherlands experienced a measles outbreak in orthodox Protestant communities with low measles–mumps–rubella vaccination coverage. Assessing total outbreak costs is needed for public health outbreak preparedness and control. Total costs of this outbreak were an estimated $4.7 million.

During May 2013–March 2014, the Netherlands was affected by a large measles outbreak (*1*). The outbreak began in the center of the country in an orthodox Protestant community and spread mainly to regions with low vaccination coverage. Overall, the Netherlands has high measles–mumps–rubella (MMR) vaccination coverage, with >95% coverage for the first dose of MMR for children. However, some orthodox Protestant and anthroposophic communities opt out of childhood vaccination programs on religious grounds or on the basis of personal beliefs (*2*). In addition to the effects of disease on a society, measles outbreaks have economic consequences, including direct medical costs and productivity losses. Moreover, a measles outbreak demands a range of responses from the National Institute for Public Health and the Environment (RIVM) and municipal public health services (MHS). Assessing outbreak costs, including costs of response activities by public health authorities, can help in planning for future outbreaks and in optimizing allocation of public resources. Recent research on measles outbreak costs in industrialized countries is scarce and has addressed hospitalizations costs (*3*), costs of imported cases of measles (*4*–*7*) or small outbreaks (*8*,*9*). We assessed the economic costs of a large measles outbreak in the Netherlands.

## The Study

All physicians and laboratories are mandated to report measles to MHSs in the Netherlands. Each MHS records patient information in a national database, which includes information on age, postal code, date of symptoms, complications, hospitalization, and source of infection. Notifications of measles cases were used to assess medical costs and productivity losses (Technical Appendix). Information on additional serologic tests and extra vaccinations among health care workers in hospitals were obtained from a study on the implementation of measles guidelines for hospitals (Technical Appendix). Information about vaccinations of infants and older unvaccinated children in response to the outbreak was retrieved from the national immunization register. We interviewed staff at MHSs and the RIVM to assess the amount of personnel time related to outbreak response activities (Technical Appendix).

During the epidemic, 2,700 measles cases were reported, mostly among children 5–14 years of age (Table 1). In 329 patients, complications such as otitis media, pneumonia, and encephalitis developed. One child died from measles complications, and 181 patients were hospitalized. One patient with encephalitis spent 8 months in a rehabilitation clinic. Of patients who consulted a physician but were not hospitalized, 199 experienced complications, mostly otitis media (104 patients) or pneumonia (75 patients). Total estimated cost for direct health care was $1,255,718 (mean $465/case). An additional $365,885 ($136/case) was attributed to productivity losses and informal child care losses (Technical Appendix Table 1). In 2013, most (85%) responding hospitals in the Netherlands offered a serologic test to employees to ensure that they were sufficiently protected against measles (Technical Appendix). Employees identified as being at risk for measles infection were offered an MMR vaccination. On average, 80 serologic tests led to 63 vaccinations per hospital for a total estimated cost of $222,203 (Technical Appendix Table 2). 

**Table 1:** Estimated direct health care costs during measles outbreak, the Netherlands, 2013–2014*

Type of cost	Total no. patients	Unit cost, $	Average health care utilization	Total cost, $
Physician consultation				
Uncomplicated measles, no. visits	2,320	37.35	0.2	17,330
Uncomplicated measles, no. phone calls	2,320	18.07	0.1	4,192
Hospitalizations, no. cases	181	37.35	1.0	6,760
Other complicated measles, no. cases	199	37.35	2.0	14,865
Treatment for pneumonia in general practice, no. cases†	75	16.02	1.0	1,202
Length of hospitalization, d				
General ward	174	600	4.6	480,240
Intensive care unit	7	2,866	13.1	262,812
Rehabilitation	1	447	245	109,515
Serologic test results, no. cases†			.	
Positive	139	21.37	1.0	2,970
Negative	854	21.37	1.0	18,250
DNA/RNA amplification, no. cases‡				
Positive	765	251.55	1.0	192,436
Negative	577	251.55	1.0	145,144
Total				1,255,718

*Costs are calculated in 2013 US dollars ($). Total number of measles cases = 2,700. Total cost differs from sum of category costs because of rounding.  †IgM. ‡PCR.

At the start of the outbreak, RIVM convened a national outbreak management team to discuss a strategy regarding targeted vaccination campaigns for infants living in communities with low vaccination coverage and for previously unvaccinated persons. A total of 6,652 infants received a complimentary MMR vaccination. Among children 18 months–19 years of age, 6,948 received an MMR vaccination during July 2013–March 2014. Costs for these vaccinations were $299,840. During this outbreak, the RIVM also coordinated outbreak control, conducted enhanced surveillance, and responded to extensive media attention (online Technical Appendix). Total costs for outbreak response activities by the RIVM were an estimated $698,280 ($259/case). In addition, we collected information from 6 MHSs that together had recorded more than half of all notified measles cases nationally. Their response activities included registration and processing of cases, vaccination activities, and advising of local authorities, professionals, and the general population (Technical Appendix). Total estimated costs for all MHSs was $1,852,470 ($686/case).

The MHSs incurred most of the costs of the outbreak, followed by costs for hospitalizations (Table 2). Costs of outbreak response activities by the RIVM were also considerable. Costs classified as other medical costs (i.e., consultations with general practitioners), productivity losses, and costs for vaccination campaigns were among the lowest (Table 2; Technical Appendix Table 3).

**Table 2:** Distribution of costs of measles outbreak, the Netherlands, 2013–2014*

Category	Costs, $	% of total costs
MHS	1,852,470	39.5
Hospitalization	852,567	18.2
RIVM	698,280	14.9
Production losses	365,885	7.8
Laboratory tests	358,801	7.6
Vaccination of children	299,840	6.4
Vaccination of health care workers	222,203	4.7
General practitioner consultation	44,350	0.9
Total	4,694,395	100

*Costs are calculated in 2013 US dollars ($). Total cost differs slightly from sum of category costs because of rounding. MHS, municipal public health services; RIVM, National Institute for Public Health and the Environment, the Netherlands.

## Conclusions

The measles outbreak occurring in the Netherlands during 2013–2014 was associated with substantial costs of ≈$4.7 million (€3.9 million), or 0.0042% of overall health care costs ($113 billion in 2013) in the Netherlands. The 2,700 reported measles cases during this outbreak resulted in an estimated $1,739 per case. Outbreak management costs were the primary cost, probably because of demands for expert advice, response to extensive media attention, registration of notified cases, and more surveillance activities than usual.

Despite being substantial, the outbreak costs in our study are underestimated. Because of data limitations, we were unable to estimate normal human immunoglobulin costs, patients’ traveling costs, or costs of vaccinations of adults or of long-term complications of disease. Also, reported cases in other countries have been linked to this outbreak, including Canada, United States, and Belgium; associated costs for cases exported to other countries are not included in our calculations. Furthermore, surveillance systems are affected by a degree of underreporting; therefore, uncertainty exists about the “true” economic costs of disease (*10*). In a previous measles outbreak in the Netherlands, the estimated true number of measles cases was ≈10 times the number of cases reported in the surveillance system (*11*). Moreover, only 47% of hospitalized cases in the previous outbreak were reported (*12*). Applying these data to our results, the estimated total outbreak costs would be ≈$0.9 million higher. Further research into the extent of underreporting in this outbreak is planned.

In Australia, the public health unit cost for responding to a single case of measles was $1,701 (*7*), a similar amount to our results. In the United States, costs of containing an outbreak were estimated at $6,180 per case. Additional US studies report that containment of a single imported measles case resulted in even higher costs per case (*5*,*6*). Explanations for the higher costs in the United States include more extensive contact tracing and higher medical care expenses.

The 2013–2014 measles outbreak posed considerable logistical challenges for MHS staff. Registration of reported cases contributed especially to the increased workload and costs created by this measles outbreak. To reduce this workload during a large outbreak, information considered to be critical for review could be collected for most patients, who usually recover within a few days or weeks, while more detailed information should continue to be collected for patients with complications or serious illness. 

Measles substantially affects patients’ quality of life (*13*) and their ability to perform their usual daily activities. Complications resulting from measles, such as pneumonia, encephalitis, and subacute sclerosing panencephalitis, sometimes occur a few years after the illness (*14*). Complications from measles also affect quality of life and incur high financial costs, as shown in the extensive rehabilitation care needed by a patient with encephalitis that resulted from this outbreak. In the Netherlands, because religious arguments affect vaccination rates (*15*), elimination of measles will be challenging. Measles outbreaks are expected to continue to cause substantial effects from disease and economic costs. To prepare for new outbreaks, medical costs, productivity losses, and containment costs should be considered. 

**Technical Appendix.** Detailed methods, results, and tables showing additional estimated economic costs for measles outbreak in the Netherlands, 2013–2014.
