# Correction: Vol. 21, No. 11

**DOI:** 10.3201/eid2201.C22201

**Published:** 2016-01

**Keywords:** errata, erratum, correction

The affiliation for Laura Nic Lochlainn was listed incorrectly in Economic Costs of Measles Outbreak in the Netherlands, 2013–2014 (A.W.M. Suijkerbuijk al.). She is with the European Programme for Intervention Epidemiology Training (EPIET) at the European Centre for Disease Prevention and Control, Stockholm, Sweden. The article has been corrected online (http://wwwnc.cdc.gov/eid/article/21/11/15-0410_article).

