# Supplementary material for: Economic Costs of Measles Outbreak in the Netherlands, 2013–2014
Source: Emerg Infect Dis. 2015 Nov;21(11):2067–9. doi: 10.3201/eid2111.150410 (PMC4622243; doi:10.3201/eid2111.150410)
Supplement: Supplementary file 1 — Technical Appendix. Detailed methods, results, and tables showing additional estimated economic costs for measles outbreak in the Netherlands, 2013–2014. [file 15-0410-Techapp-s1.pdf]

Article DOI: http://dx.doi.org/10.3201/eid2111.150410

Economic Costs of Measles Outbreak in the Netherlands, 2013–2014

Technical Appendix

Additional Methods, Results, and Tables

Case Definition

The measles case definition used is based on the presence of clinical symptoms and laboratory confirmation of diagnosis. An epidemiologically linked case is defined as someone with a matching clinical presentation who had contact with a laboratory-confirmed case. The measles case definition used is consistent with that of the European Centre for Disease Control and Prevention (*1*).

Assessment of Outbreak Costs

Direct Medical Costs

Information on health care use regarding the number of general practitioner (GP) visits due to measles infection has been reported (*2*). In a previous Dutch outbreak, van Isterdael et al. (2004) estimated that 30% of the measles patients consulted their GP. We used this average number of consultations for patients without complications and assumed that 1 third of the patients contacted their GP by phone and the remainder by a GP visit (unpub. data, Tom Woudenberg, RIVM, Bilthoven, the Netherlands). In line with the Dutch health care system, we assumed that all hospitalized patients had consulted a GP once. Patients for whom complications developed (but who were not hospitalized) were assumed to have visited their GP twice. According to Dutch guidelines, apart from pneumonia, most measles complications reported by GPs (i.e., otitis media, dehydration, and upper respiratory infection) do not require further treatment (*3*). Medical costs were gathered from standard unit cost lists (*4*) and list prices available online (*5*). The unit cost per hospitalized day comprises treatment in hospital, treatment in an intensive care unit, and clinician consultation fees. Because the database of notified cases did not include negative diagnostic tests, we estimated the total number of diagnostic tests by applying the ratio of positive and negative diagnostic tests of the RIVM laboratory to all positive tests recorded in the national database.

All costs are expressed as U.S. dollars as of 2013. Euros were converted to US$ by using data on purchasing power parity of the Organization for Economic Co-operation and Development: 1 US$ = 0.83 euro.

Productivity Losses

Almost all notified measles cases were unvaccinated orthodox Protestants. Orthodox Protestants constitute a Calvinistic religious minority in the Netherlands who believe in predestination and divine providence. Their lifestyle is based on the scripture and religion, which play an important role in their daily life. Many of them reject vaccination for religious reasons. Because most orthodox Protestant women do not have a paid job and stay at home to take care of their children, productivity losses of women with measles were calculated as loss of informal child care (*4*,*6*). To calculate productivity losses for men, we used standard tariffs (mean, all ages) (*4*), adjusted for work participation in all age groups (*7*). We did not calculate productivity losses for parents taking care of sick children. The duration of productivity losses was calculated for the average duration of illness (i.e., 14 days).

Costs of Targeted Vaccination Campaigns

In the beginning of the outbreak, health care workers born after 1965 were encouraged to check their vaccination and measles infection status and complete their measles–mumps–rubella (MMR) vaccination if necessary (*8*). Data about serologic tests and extra vaccinations among health care workers in hospitals were obtained from a study on the implementation of measles guidelines for hospitals (unpub. data, Lydia Fievez, RIVM). In this study, 85% (69) of responding hospitals (81 of 88 hospitals in the Netherlands) offered a serologic test to employees.

In July 2013, parents of children 6–14 months of age who lived in communities with vaccination coverage <90% or who belonged to orthodox Protestant communities received a personal invitation for an early MMR vaccination. The normal schedule for MMR in the Netherlands is to receive the MMR-1 at 14 months and the MMR-2 at 9 years of age. Children 6–12 months of age received an MMR-0 vaccination, and those 12–14 months of age received an early MMR-1 vaccination. In addition, the municipal public health services (MHSs) offered an MMR vaccination to children and adolescents within the vaccination program up to 19 years of age if they had not yet received an MMR vaccination. To avoid including routine MMR-1 vaccinations at 14 months in the outbreak costs, we selected children who received their MMR-1 during the outbreak period who were >18 months–19

years of age. All vaccinations used in this study were recorded in the national immunization register (unpub. data; Praeventis database for registering vaccinations in the Netherlands). Vaccine price and administration costs were gathered from the Dutch Healthcare Authority (*5*).

Costs of Outbreak Response Coordination at the National Level (RIVM)

Personnel time spent on outbreak control and on investigating and processing the outbreak was also estimated. Given the limited resources, this reallocation of personnel time represents the loss of other production (i.e., opportunity costs). Personnel time of the RIVM was determined by hours allocated to surveillance, response, laboratory, and vaccination activities at the national level. This time estimate was obtained from personal interviews with the personnel in the relevant departments within RIVM. We calculated personnel costs by multiplying a person’s salary tariff by the time spent on the measles outbreak.

Costs of Outbreak Response Coordination at the Regional Level (MHS)

To estimate the amount of personnel time associated with local outbreak response activities, we developed a questionnaire for semistructured interviews of MHS staff (i.e., doctors, nurses, and managers) in some of the regions with the highest number of notified measles cases. The interviews explored all MHS activities and the associated time investment of the personnel involved. All possible local reports and registries were collected for additional information. The estimated time investment of physicians, nurses, nursing assistants, managers, and communication employees involved in the outbreak were converted to costs by using an average salary tariff per hour of MHS staff. We calculated time and costs per notified case in these regions and extrapolated these estimates to all notified cases in the Netherlands.

Additional Results of Costs of Outbreak Response Coordination

Costs of Outbreak Response Coordination at the National Level (RIVM)

Four departments at RIVM were involved with the measles outbreak: the Centres for Epidemiology and Surveillance; Communicable Disease Control; Infectious Diseases Research Diagnosis and Screening; and Policy and Regional Support. During the outbreak, representatives of these departments participated in a weekly response meeting at which the current outbreak and national containment strategies were discussed. Technical Appendix Table 4 shows the total labor time and costs for all personnel involved. Total costs were estimated at $698,280.

The interviewed MHS staff confirmed that measles response activities were time consuming, especially registration and processing of new measles cases in their region. On average, these activities required 2–3.5 hours per case. At the beginning of the outbreak, numerous internal staff meetings were held to organize regional response activities adequately. Vaccination activities were limited because the targeted group of orthodox Protestants is generally unwilling to accept measles vaccination. Of the different personnel categories, public health nurses spent most of their time performing outbreak response activities (Technical Appendix Table 5). Based on these data, the total cost for all MHSs was estimated to be $1,852,470 ($686.1 for each of 2,700 notified cases).

References

1. Commission of the European Communities. Commission decision of 28/IV/2008 amending decision 2002/253/EC laying down case definitions for reporting communicable diseases to the community network under decision No 2119/98/EC of the European Parliament and of the Council. 2008 [cited 2015 Mar 3]. http://ec.europa.eu/health/ph_threats/com/docs/1589_2008_en.pdf

2. van Isterdael CE, van Essen GA, Kuyvenhoven MM, Hoes AW, Stalman WA, de Wit NJ. Measles incidence estimations based on the notification by general practitioners were suboptimal. J Clin Epidemiol. 2004;57:633–7. PubMed http://dx.doi.org/10.1016/j.jclinepi.2003.11.012

3. van Steenbergen JE, Timen A, Beaujean DMJA. LCI measles guideline [in Dutch]. 2014 [cited 2015 Mar 3]. http://rivm.nl/Documenten_en_publicaties/Professioneel_Praktisch/Richtlijnen/Infectieziekten/LCI_richtlijne/LCI_richtlijn_Mazelen_morbilli

4. Hakkaart-van Roijen L, Tan SS, Bouwmans CAM. Manual for Cost Research, Methods and Standard Cost Prices for Economic Evaluations in Health Care [in Dutch] Diemen (the Netherlands): College voor Zorgverzekeringen; 2010.

5. Dutch Health Care Authority. Cost of vaccines [in Dutch]. Beleidsregel CA-300-567. [cited 2015 Mar 3]. http://www.nza.nl/1048076/1048090/CA_300_567__Kosten_vaccins.pdf

6. Baars-Blom J. Girls living in orthodox Protestant communities [in Dutch]. Kampen (the Netherlands): Kok; 2006.

7. Statistics Netherlands. Employed labor and working hours [in Dutch]. 2015 [cited 2015 Mar 3]. http://statline.cbs.nl/Statweb/publication/?DM=SLNL&PA=82647ned&D1=a&D2=1-2&D3=0,13&D4=49-61&HDR=G3&STB=G1,G2,T&VW=T/

8. RIVM. Guideline for prevention of measles in health care [in Dutch]. 2013 [cited 2015 Mar 3]. http://www.rivm.nl/Documenten_en_publicaties/Professioneel_Praktisch/Richtlijnen/Infectieziekten/LCI_richtlijnen/LCI_richtlijn_Mazelen_morbilli/Download/Advies_bescherming_tegen_mazelen_in_de_gezondheidszorg

Technical Appendix Table 1. Estimated indirect costs and productivity losses for men and women during measles outbreak, the Netherlands, 2013–2014*

	Sex	
Category	M	F
Adult cases, no.	100	109
Employment or provision of informal child care, %	72.3%	100%
Employment or provision of informal child care, h/wk	36.1	40.0
Productivity costs/h, $†	42.67	16.42
Total productivity losses, $†	222,740	143,145

*Productivity losses were calculated for 2 weeks (10 working days) as average duration of illness. Because orthodox Protestant women tend to stay at home taking care of their children, we calculated production losses of work absenteeism for men and production losses of informal child care provided by women.
†Production costs and losses are calculated in 2013 US dollars ($).

Technical Appendix Table 2. Costs of targeted vaccination campaigns during measles outbreak, the Netherlands, 2013–2014*

	Costs and factors affecting costs			
Population/category of cost	Unit costs, $	Hospitals, no.	Average no.	Total costs, $
Health care workers				
Serologic test	21.37	69	80	117,962
Vaccination	8.33	69	63	36,211
Administration costs	15.65	69	63	68,031
Total				222,203
Children 6–14 mo		MMR-0, no.	Early MMR-1, no.	
Vaccination	8.33	5,238	1,414	55,411
Administration costs	14.76	5,238	1,414	98,184
Total				153,595
Children 18 mo–19 y			MMR-1, no.	
Vaccination	8.33		6,948	57,877
Administration costs 0–5 y	14.76		2,764	40,797
Administration costs 5–19 y	11.37		4,184	47,572
Total				146,246

*Costs are calculated in 2013 US dollars ($). Total cost may differ from sum of category costs because of rounding. MMR, measles–mumps–rubella; MMR-0, extra MMR vaccination given to children 6–12 months of age; Early MMR-1, MMR vaccination given to children 12–14 months of age; MMR-1, MMR vaccination given to children 18 months–19 years of age.

Technical Appendix Table 3. Main cost categories of measles outbreak, the Netherlands, 2013–2014*

Category	Costs, $	% of total costs
Outbreak management	2,550,750	54.3
Medical costs	1,255,718	26.8
Prevention (vaccination)	522,044	11.1
Production losses	365,885	7.8
Total	4,694,395	100

*Costs are calculated in 2013 US dollars ($).Total cost differs slightly from sum of category costs because of rounding.

Technical Appendix Table 4. Labor time and costs for personnel involved in outbreak management at the national level (RIVM) during the outbreak of measles, the Netherlands, 2013–2014*

Department	Task	Labor time, h	Costs, $
Disease control	Coordination of outbreak control and communication	2,730	300,723
Support	Organization of MMR-0 and MMR-1 vaccination campaigns	1,754	177,372
Surveillance	Analysis and reporting of outbreak data	996	118,257
Diagnostics	Advice and Interpretation of laboratory results†	846	101,928
Total			698,280

*Costs are calculated in 2013 US dollars ($). RIVM, Ministry of Health, Welfare and Sport, the Netherlands; MMR, Measles–Mumps–Rubella. MMR-0, extra MMR vaccination given to children 6–12 months of age; Early MMR-1,MMR vaccination given to children 12–14 months of age; MMR-1, MMR vaccination given to children 18 months to 19 years of age.
†Costs of laboratory tests are presented in Table1 of article.

Technical Appendix Table 5. Labor time and costs for personnel involved in outbreak management at regional level MHSs in measles outbreak, the Netherlands, 2013–2014*

Employee	Tariff, $	Labor time per notified case, h	Costs per notified case, $
Nurse	69	5.2	359.00
Physician	107	1.9	203.00
Manager	103	0.6	62.00
Communication employee	88	0.4	35.00
Nursing assistant	54	0.5	27.00
Total		8.6	688.00

*Costs are calculated in 2013 US dollars ($).Total cost differs slightly from sum of category costs because of rounding.
